# A Dual-Energy CT Radiomics of the Regional Largest Short-Axis Lymph Node Can Improve the Prediction of Lymph Node Metastasis in Patients With Rectal Cancer

**DOI:** 10.3389/fonc.2022.846840

**Published:** 2022-06-07

**Authors:** Dongqing Wang, Zijian Zhuang, Shuting Wu, Jixiang Chen, Xin Fan, Mengsi Liu, Haitao Zhu, Ming Wang, Jinmei Zou, Qun Zhou, Peng Zhou, Jing Xue, Xiangpan Meng, Shenghong Ju, Lirong Zhang

**Affiliations:** ^1^Department of Medical Imaging, The Affiliated Hospital of Jiangsu University, Zhenjiang, China; ^2^School of Medicine, Jiangsu University, Zhenjiang, China; ^3^Department of General Surgery, The Affiliated Hospital of Jiangsu University, Zhenjiang, China; ^4^School of Medicine, Southeast University, Nanjing, China; ^5^Department of Radiology, Zhongda Hospital, Southeast University, Nanjing, China

**Keywords:** rectal cancer (RC), lymph node metastasis, radiomics, dual-energy scanned projection, machine learning, clinical prediction rule

## Abstract

**Objective:**

To explore the value of dual-energy computed tomography (DECT) radiomics of the regional largest short-axis lymph nodes for evaluating lymph node metastasis in patients with rectal cancer.

**Materials and Methods:**

One hundred forty-one patients with rectal cancer (58 in LNM+ group, 83 in LNM- group) who underwent preoperative total abdominal DECT were divided into a training group and testing group (7:3 ratio). After post-processing DECT venous phase images, 120kVp-like images and iodine (water) images were obtained. The highest-risk lymph nodes were identified, and their long-axis and short-axis diameter and DECT quantitative parameters were measured manually by two experienced radiologists who were blind to the postoperative pathological results. Four DECT parameters were analyzed: arterial phase (AP) normalized iodine concentration, AP normalized effective atomic number, the venous phase (VP) normalized iodine concentration, and the venous phase normalized effective atomic number. The carcinoembryonic antigen (CEA) levels were recorded one week before surgery. Radiomics features of the largest lymph nodes were extracted, standardized, and reduced before modeling. Radomics signatures of 120kVp-like images (Rad-signature_120kVp_) and iodine map (Rad-signature_Imap_) were built based on Logistic Regression *via* Least Absolute Shrinkage and Selection Operator (LASSO).

**Results:**

Eight hundred thirty-three features were extracted from 120kVp-like and iodine images, respectively. In testing group, the radiomics features based on 120kVp-like images showed the best diagnostic performance (AUC=0.922) compared to other predictors [CT morphological indicators (short-axis diameter (AUC=0.779, IDI=0.262) and long-axis diameter alone (AUC=0.714, IDI=0.329)), CEA alone (AUC=0.540, IDI=0.414), and normalized DECT parameters alone (AUC=0.504-0.718, IDI=0.290-0.476)](*P*<0.05 in Delong test). Contrary, DECT iodine map-based radiomic signatures showed similar performance in predicting lymph node metastasis (AUC=0.866). The decision curve showed that the 120kVp-like-based radiomics signature has the highest net income.

**Conclusion:**

Predictive model based on DECT and the largest short-axis diameter lymph nodes has the highest diagnostic value in predicting lymph node metastasis in patients with rectal cancer.

## Introduction

Colorectal cancer is the most common gastrointestinal tumor and the third most diagnosed cancer in men and women. It has the second-highest mortality rate after lung cancer (1). Rectal cancer accounts for more than one-third of colon cancer cases (2). The occurrence of lymph node (LN) metastasis in patients with rectal cancer is highly correlated with poor clinical prognosis and tumor recurrence (2). Yet, different lymph node staging obtained by preoperative imaging evaluation may lead to different clinical decisions and, consequently, different treatment options (3). For example, surgery is usually recommended for patients with N0 (no regional LN metastasis), while preoperative neoadjuvant therapy is often used for those with N1 (1–3 regional LNs metastasis) or N2 (4 or more LNs metastasis).

So far, various traditional imaging modalities, including ultrasound, computed tomography (DECT), magnetic resonance (MR) imaging, and positron emission tomography (PET), have been applied to analyze the lymph node metastasis in patients with rectal cancer; yet, none of them have satisfactory diagnostic performance. Moreover, the diagnosis of LN status relies on their size and the reader’s subjective judgment (4, 5). Recently, Gao et al. assessed the methodological and reporting quality of systematic reviews that evaluated the diagnostic value of four different imaging modalities (CT, endorectal ultrasonography (ERUS), endoscopic ultrasound (EUS), and MRI) for LN involvement in patients with rectal cancer. He concluded that no modality was particularly accurate (6). For example, preoperative high-resolution MR shows high soft-tissue resolution and can improve the accuracy of preoperative staging of rectal cancer; yet, its accuracy in detecting N staging is lower than 60% (7, 8). Moreover, no standard criteria for LN evaluation have been proposed so far. For example, some studies emphasized the importance of LN morphological predictors (8), while others disagreed with this data (9).

DECT is an emerging imaging technology (10) used to obtain mixed-energy images, single-energy images, or separate base material images through instantaneous switching of the tube or dual-tube. This feature enables selective quantification of different image materials with different electron density characteristics and atomic numbers, creating material-specific image datasets. The iodine map of DECT, which represents the iodine content in tumor tissues, has been considered a powerful tool for tumor diagnosis and characterization (11). Iodine maps and spectral CT have been useful in assessing rectal cancer by displaying more lesions with higher sensitivity (12) and distinguishing lesions from intestinal contents (13). In terms of LN, previous studies have suggested that quantitative parameters such as normalized iodine concentration (NIC) and effective atomic number (Z_eff_) can be used to evaluate the LN status of rectal cancer (14). However, these studies only measured the average value of the whole lymph node on DECT while ignoring a large amount of heterogeneous texture and morphological information.

Radiomics is a relatively new quantitative approach to medical imaging. It uses characterization algorithms to extract quantitative features from medical images, such as shape features, intensity-based statistical features, texture features and so on (15, 16). Some studies have suggested that radiomic analysis of rectal tumor images might improve the prognostic evaluation of the tumor and the patients’ characterization. For example, Huang et al.(17) found that radiomic score (rad-score) of primary lesions combined with clinical feature nomogram based on readers’ subjective evaluation of nodules can improve the accuracy of detecting LN metastasis in patients with colorectal cancer. Yet, the final performance of the model was poor (AUC=0.778). In addition, the study did not examine the regional lymph nodes.

Based on the data reported above, we summarized three main deficiencies in the imaging evaluation of lymph nodes in rectal cancer: (1) the diagnostic criteria and methods for assessing LN, which have not yet reached a consensus. There is no clear threshold for lymph node size and other indicators for reference. (2) The repeatability of some ambiguous subjective signs has not been yet verified, which reduces the accuracy of the diagnosis. (3) Lack of quantitative and heterogeneous analysis of lymph nodes, especially on images with high spatial resolution and thin slices, such as DECT. Thus, in this study, we further explored the value of DECT radiomics of the regional lymph nodes for evaluating lymph node metastasis in patients with rectal cancer.

## Materials and Methods

### Patients

**Figure 1:**
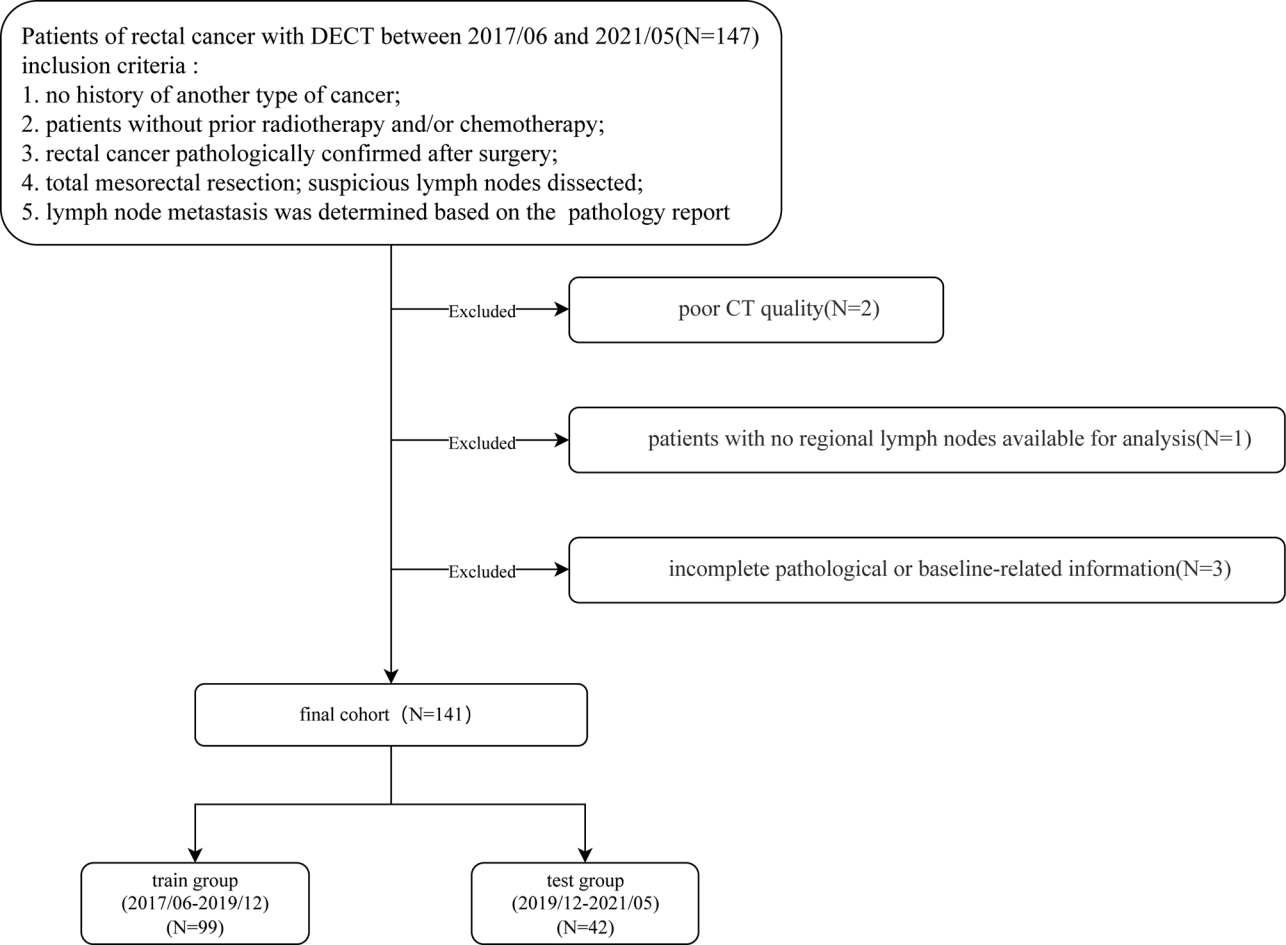
Patient selection flowchart.

Patients diagnosed with rectal cancer by colonoscopy who underwent a spectral CT (a kind of DECT) scan for preoperative evaluation between June 2017 to May 2021 were included in this study. The inclusion criteria were: 1. no history of another type of cancer; 2. patients without prior radiotherapy and/or chemotherapy; 3. rectal cancer pathologically confirmed after surgery; 4. total mesorectal resection; suspicious lymph nodes were dissected; 5. lymph node metastasis was determined based on the final pathology report of the surgical specimen. Exclusion criteria were: 1. poor CT image quality; 2. patients with no regional lymph nodes available for analysis; 3. incomplete pathological or baseline-related information (**Figure 1**).

Finally, 141 patients (101 males, 40 females; median age 67, IQR: 58-73; range: 32-87 years old) were included in the study. The T tumor stage was recorded according to the guideline of NCCN (18). The carcinoembryonic antigen (CEA) index level was recorded one week before surgery. LNM+ patients were defined as those having one or more lymph node metastasis (identified by the pathologists); otherwise, patients were classified as LNM-. According to the examination time of DECT, the patients were assigned to the training and testing groups using a ratio of 7:3. Splitting patients by examination time is called temporal validation, which is recommended by many experts (19, 20).

The institutional review board approved this prospective study, and all patients signed an informed consent form.

### CT Reconstruction and Post-Processing

All patients underwent head-first scanning in the supine position. Before the exam, patients fasted for 6 hours without undergoing bowel cleansing preparation. Patients were asked to hold their breath during the scanning. DECT (Revolution CT, GE Healthcare, USA) with Gemstone Spectral Imaging (GSI) mode was used to perform a plain scan and enhanced scan (from the diaphragm to the lower edge of the pubic symphysis). The non-ionic contrast agent ioversol (350 mg/ml, Jiangsu Hengrui Pharmaceutical Co., Ltd., China; 1.5ml/Kg, injection rate: 3.0 or 3.5ml/s) was applied for enhancement by the nursing staff. Then, 30ml of normal saline was injected intravenously at the same rate. The spectral scanning parameters were: the tube voltage was instantaneously switched between 140kVp and 80kVp, the tube current was set to the automatic tube current, and the maximum reference tube current was 600mA; the tube rotation speed was 0.5 sec/r; the pitch was set to 1.984:1. The 120kVp-like image in the venous phase was automatically reconstructed according to the kVp-like kernel function after scanning, and the thickness was 1.25mm. The CT data were reconstructed using 50% adaptive statistical iterative reconstruction (ASIR, GE Healthcare) and then transferred to an advanced workstation (AW4.7, GE Healthcare) for analysis and post-processing.

Raw data were loaded to the GSI Viewer software application, after which an iodine (water) map was obtained. Iodine concentration was 100mg/cm^3^. Since the 120kvp-like and iodine maps were reconstructed from the same raw data, the layer thickness, spacing, and spatial position information were the same (no registration was required).

### Selection of the Largest Regional Lymph Node

On the 120kVp-like venous phase image, lymph nodes inside and outside the mesorectum along the superior rectal artery were examined. The areas included the mesorectum and the anterior sacrum. The largest lymph node was defined as the lymph node with the longest short-axis diameter for the superior rectal artery and the lateral mesangial inner iliac lymph nodes. If there were multiple large lymph nodes with similar diameters (the difference is ≤1mm), the following classification criteria were applied: 1. lymph nodes that are heterogeneously enhanced, including necrosis or mucinous texture; 2. lymph nodes that are round rather than oval; 3. lymph nodes that are closer to the lesion; 4. lymph nodes that are located above but not below the lesion.

The above annotation process was not repeated for arterial phase images or iodine maps, but the lymph node determined by the venous phase was searched and matched to the corresponding position.

All lymph nodes were analyzed by two experienced radiologists (Zhou Q. and Zou J.M. with more than 10 years of experience in abdominal imaging diagnosis) who were blind to the postoperative pathological results. A third radiologist (Zhang L.R., with more than 20 years of experience in abdominal imaging diagnosis) was invited if there were any disagreements. The two readers manually measured the long and short-axis diameter of the selected lymph node using the workstation on the 120 kVp-like axial images, and the average of the measured values of the two was recorded.

### Quantitative Parameters of DECT for Evaluating Largest Regional Lymph Nodes

This method was performed as previously described (21). The whole measurement process was carried out in the GSI VIEWER of the workstation. Two readers (Zhou Q. and Zou J.M.) manually drew ROI on the cross-sectional image to cover the entire lymph node as much as possible, excluding the surrounding mesangial tissue. ROI was also placed on the descending aorta at the bifurcation of the right renal artery. The iodine concentration (IC) and effective atomic number (Z_eff_) of the largest lymph nodes and aorta in the arterial and portal phases were obtained. The normalized iodine concentration (NIC) value is the iodine uptake value of the lymph nodes divided by the iodine uptake value of the aorta.


$$NIC_{LN}=IC_{LN}/IC_{aorta}$$

The normalized Z_eff_ is the effective atomic number of the lymph node divided by the effective atomic number of the aorta.


$$NZ_{eff_{LN}}=Z_{eff_{LN}}/Z_{eff_{aorta}}$$

Four DECT quantitative parameters included: AP NIC, AP normalized Z_eff_, VP NIC, and VP normalized Z_eff_. The above parameters were analyzed by averaging the measured values of the two readers.

### Radiomics Feature Extraction

**Figure 2:**
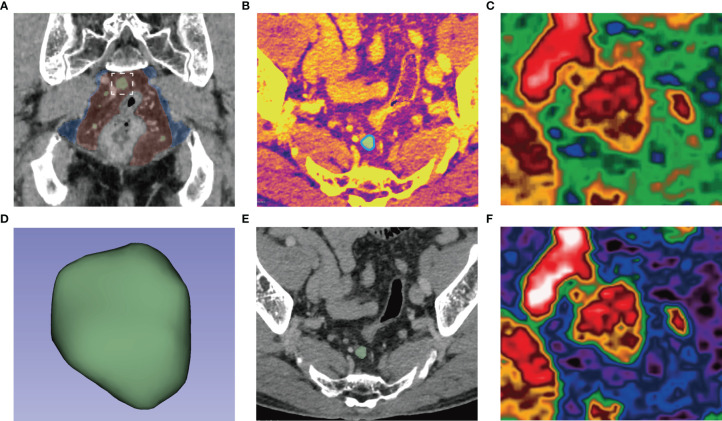
**(A)** After searching for lymph nodes in the mesorectum (red) and extramesenteric (blue) areas, the largest lymph nodes (white box) were delineated along its edge (blue line) in the axial iodine map **(B)** to form a 3D-ROI **(D)**. The ROI could be used for 120kVp-like images without registration **(E)**. The pseudocolor map of the largest lymph nodes in the iodine map **(C)** and 120kVp-like image **(F)** show apparent internal heterogeneity.

All segmentation was performed on 3D Slicer v. 4.8.1. A reader (Zou J.M.) outlined all the slices of the largest lymph node on the venous iodine map. Thirty cases were randomly selected, and two radiologists (Zou J.M. and Zhou Q.) redrew their ROIs one month later for the repeatability test of features. The obtained mask was also suitable for 120kVp-like images in the venous phase. (**Figure 2**) Using pyradiomics for feature extraction based on 3D ROIs, 833 features were extracted from 120kVp-like and iodine images. The specific features are shown in **Table 1**. Intraclass correlation coefficient (ICC) was used for analyzing the consistency at intraobserver and interobserver; the features of *r* value greater than 0.7 were analyzed in the follow-up.

**Table 1:** The list of the radiomics features.

Feature category	Feature number
original	
shape	14
first order	18
GLCM	22
GLRLM	16
GLSZM	16
NGTDM	5
GLDM	14
wavelet	
LLH	91
LHL	91
LHH	91
HHL	91
HLL	91
HLH	91
HHH	91
LLL	91
**TOTAL**	**833**

GLCM, gray-level co-occurrence matrix; GLRLM, gray-level run-length matrix; GLSZM, gray-level size zone matrix; NGTDM, neighbourhood gray-tone difference matrix; GLDM, gray level dependence matrix; L, lowpass filters; H, highpass filters. In bold: "TOTAL" is just the sum of feature numbers.

### Selection of Radiomics Features and Establishment of the Models

**Figure 3:**
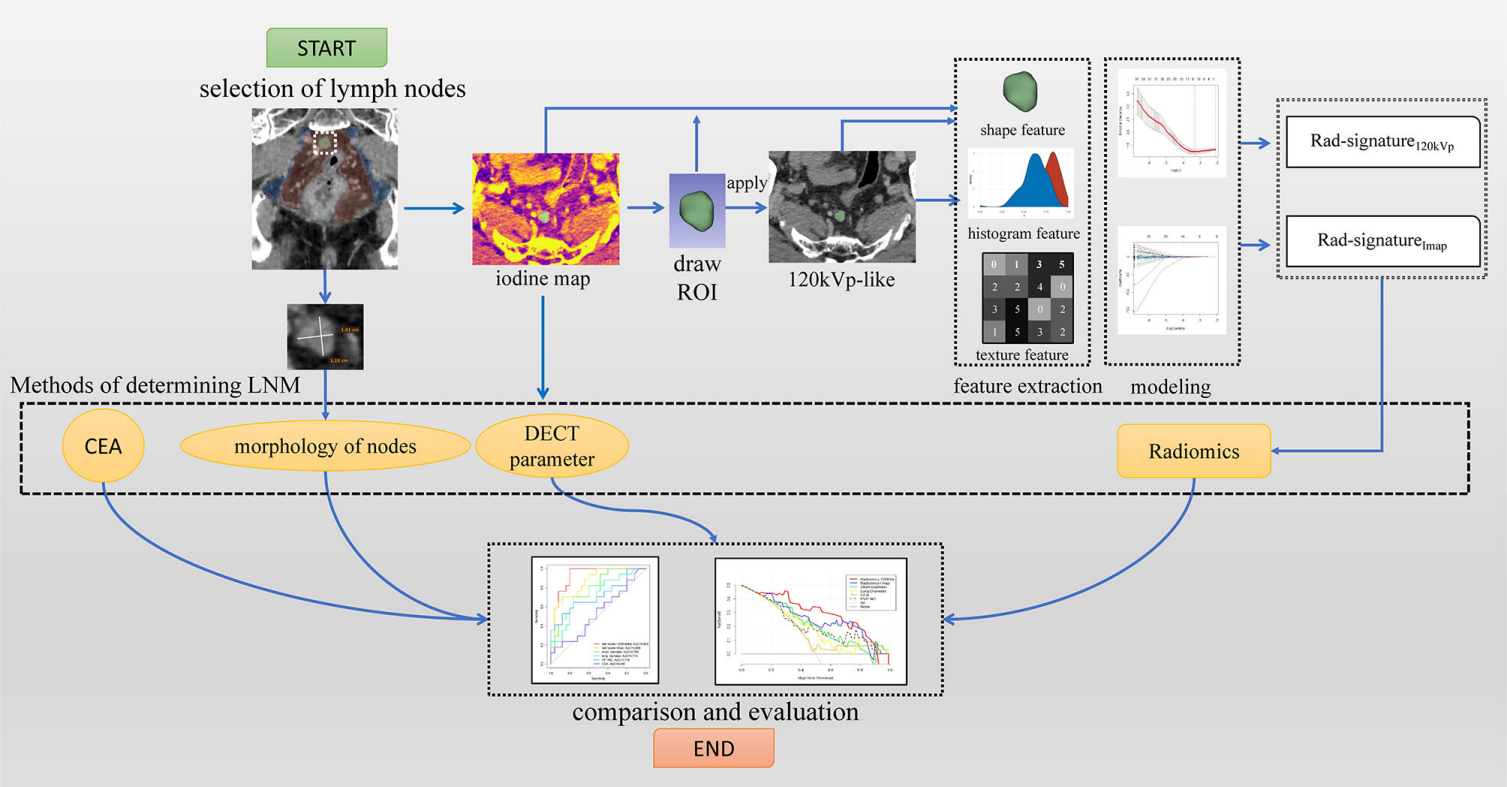
A study workflow. Imaging processing began by selecting the largest lymph nodes; finally, four categories of methods were evaluated.

This part was completed on the software FAE v3.7.0 (20). All models were built based on the same training group, and all features were standardized to a standard normal distribution using Z-score to reduce the difference in the range of feature values. Pearson correlation coefficients (PCC) were used to reduce the dimensionality of features. When the coefficient was >0.86, one of them was randomly removed for dimensionality reduction. Then recursive feature elimination (REF) was used to filter features; Logistic Regression *via* Lasso was used as a classifier for modeling. To avoid overfitting, each feature requires at least 10-15 patients to participate in the radiomic signature (22, 23). The maximum number of features was limited to 10 because the number of patients in the training group was 99. Then 10-fold cross-validation was used to select the best model based on the means of AUCs. Finally, a radiomic model of the regional largest lymph node (Rad-signature_120kVp_) based on venous phase images of 120kVp-like and a radiomics model of regional largest lymph node based on venous phase iodine maps (Rad-signature_Imap_), were established. The flow chart of the whole research scheme is shown in **Figure 3**.

### Statistical Analysis

For continuous variables, the Kolmogorov-Smirnov was used to compare the cumulative distributions of the data sets. The mean ± standard deviation was used to express normal distribution, and the T-test was used to compare the differences between groups with normal distribution; the median and quartile were used for the values that did not meet the normal distribution, and the Mann-Whitney U test was used to compare the differences between these groups.

The Chi-square test or Fisher’s exact test was used for categorical variables. The receiver operator characteristic (ROC) curves were drawn for each model or indicator, and the area under the Curve (AUC) was used to measure diagnostic performance. The Delong test was used to compare the differences in ROC curves. The point with the largest Youden index in each ROC curve was selected as the optimal threshold of the model. The Youden index, sensitivity, specificity, and accuracy were calculated. The integrated discrimination improvement (IDI) value of two radiomics signature to other predictors were computed. Finally, a decision curve was used for different models or predictors in the testing group to analyze their clinical applicability.

The radiomic signatures and the long-axis and short-axis diameter of the lymph node were used, and the Spearman correlation analysis to evaluate the correlation between the established radiomic signatures and the lymph node size index. Statistical analyses were performed using SPSS software 26.0, MedCalc 20.0, and R software 3.6.1. A *P*-value<0.05 was considered to be statistically significant.

## Results

### Patient Characteristics

A total of 141 patients with rectal cancer were included in this study. The characteristics of the patients in the training cohort and testing cohort are shown in **Table 2**. There were 99 patients in the training group and 42 in the testing group. There were 58 patients (41.13%) in the LNM+ group and 83 (58.87%) in the LNM- group. Clinical characteristics (gender, age, long-axis diameter, and short-axis diameter of the largest regional lymph node, pathological T stage, CEA, and lymph node metastasis) were not statistically different between the training group and the testing group (all *P*>0.05); while the long-axis diameter, short-axis diameter, and T stage of the lymph node were statistically different between the LNM+ group and the LNM- group (all *P*<0.05), in both training and the testing groups. Moreover, CEA was statistically different between patients with different N stages in the training group (**Table 3**).

**Table 2:** Study sample demographics and clinical characteristics.

Characteristics	Training group (n=99)	Testing group (n=42)	*P*
Sex, No. (%)			0.208
male	74 (74.7%)	27 (64.3%)	
female	25 (25.3%)	15 (35.7%)	
Age (IQR)	67 (57–73)	67.5 (58-72.25)	0.787
Long diameter, mm (IQR)	7.85 (6.06-9.88)	7.03 (5.69-9.45)	0.668
Short diameter, mm (IQR)	5.68 (4.64-8.02)	5.56 (4.39-7.19)	0.573
T stage			0.971
1	10 (10.1%)	5 (11.9%)	
2	27 (27.3%)	10 (23.8%)	
3	53 (53.5%)	23 (54.8%)	
4	9 (9.1%)	4 (9.5%)	
CEA, ng/ml (IQR)	3.91 (2.18-7.17)	3.47 (1.92-7.85)	0.690
LNM, No. (%)			0.918
positive	41 (41.4%)	17 (40.5%)	
nagetive	58 (58.6%)	25 (59.5%)	

IQR, interquartile range.

**Table 3:** Study sample demographics and clinical characteristics of patients with LNM+ and LNM- rectal cancer.

Characteristics	Training group		*P*	Testing group		*P*
	LNM+	LNM-		LNM+	LNM-
Sex, No. (%)			0.525			
male	32 (78%)	42 (72.4%)		13 (76.5%)	11 (44%)	0.174
female	9 (22%)	16 (27.6%)		4 (23.5%)	14 (56%)	
Age (IQR)	66 (59.5-73)	68 (55.75-73.25)	0.991	68.53 ± 9.04	64.52 ± 9.55	0.176
Long-axis diameter, mm(IQR)	8.84 (6.73-11.7)	7.15 (5.53-8.98)	0.002	8.74 (6.87-13.1)	6.88 (5.44-8.02)	0.02
Short-axis diameter, mm(IQR)	7.29 (5.25-10.28)	5.06 (4.21-6.29)	<0.001	6.8 (5.28-9.88)	4.97 (3.89-6.08)	0.002
T stage, No. (%)			0.005			<0.001
1	0 (0%)	10 (17.2%)		0 (0%)	5 (20%)	
2	8 (19.5%)	19 (32.8%)		0 (0%)	10 (40%)	
3	27 (65.9%)	26 (44.8%)		13 (76.5%)	10 (40%)	
4	6 (14.6%)	3 (5.2%)		4 (23.5%)	0 (0%)	
CEA, ng/ml(IQR)	4.53 (3.42-11.08)	3.31 (1.93-5.51)	0.003	3.69 (1.94-9.51)	3.2 (1.88-7.86)	0.663

IQR, interquartile range.

#### The Efficiency of Long-Axis and Short-Axis Diameter and CEA in Predicting LN Metastasis

**Figure 4:**
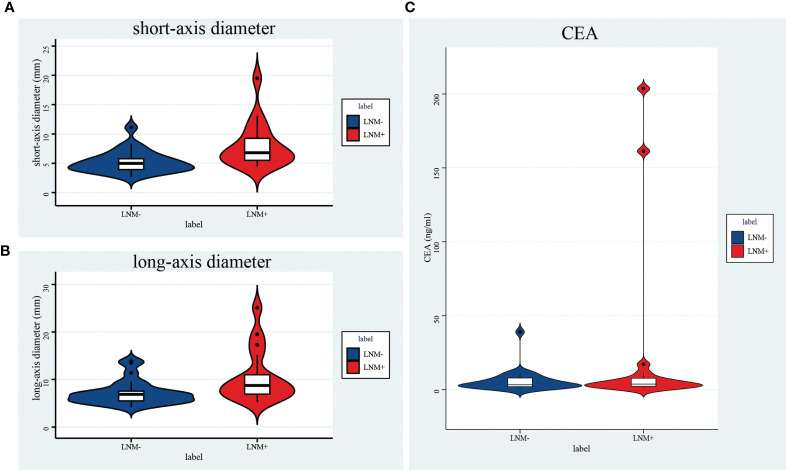
Violin plot of a short-axis diameter **(A)**, a long-axis diameter **(B)**, and CEA **(C)**. All of them were significantly different between LNM+ and LNM- groups.

The median value of the long-axis diameter of the largest lymph nodes was 7.57 (IQR: 5.88-9.73), while the median value for short-axis diameter was 5.62 (IQR: 4.63-7.66). For LNM- patients, the median value of a long-axis diameter was 7.00 (IQR: 5.52-8.89) and 4.99 (IQR: 4.02-6.28) for the short-axis diameter; for patients with LNM+, the median value of the long-axis diameter was 8.79 (IQR: 6.81-11.60), and the median value of the short-axis diameter was 7.05 (IQR: 5.27-10.10)(**Table 4**, **Figures 4A, B****)**; the difference in the long-axis diameter and short-axis diameter between patients with LNM- and LNM+ was statistically significant (*P*<0.001).

**Table 4:** Comparison of morphology predictors, CEA and DECT quantitative parameters on distributions, AUCs, cut-offs, sensitivities and specificities.

Predictor	Overall	LNM-	LNM+	*P*	AUC	CUT-OFF	Sensitivity	Specificity
long-axis diameter, mm	7.57 (5.88-9.73)	7.00 (5.52-8.89)	8.79 (6.81-11.60)	<0.001	0.691 (0.608-0.766)	6.45	62.1	80.7
short-axis diameter, mm	4.99 (4.02-6.28)	4.99 (4.02-6.28)	7.05 (5.27-10.10)	<0.001	0.755 (0.676-0.824)	8.19	60.3	71.1
APNIC	0.1786 (0.1425-0.2323)	0.1889 (0.1530-0.2448)	0.1710 (0.1374-0.2128)	0.053	0.596 (0.510-0.678)	0.1760	60.4	60.3
VPNIC	0.6622 (0.5535-0.7604)	0.6937 (0.6036-0.7800)	0.6288 (0.4715-0.7303)	0.002	0.655 (0.536-0.702)	0.5545	84.3	41.4
APNZ_eff_	0.7512 (0.7247-0.7843)	0.7563 (0.7233-0.7858)	0.7494 (0.7280-0.7777)	0.56	0.596 (0.443-0.613)	0.7537	53	58.6
VPNZ_eff_	0.9458 (0.9195-0.9656)	0.9530 (0.9311-0.9676)	0.9421 (0.9057-0.9595)	0.014	0.622 (0.536-0.702)	0.9551	48.2	70.7
CEA, ng/ml	3.90 (2.17-7.58)	3.35 (1.94-6.04)	4.42 (2.94-10.83)	0.008	0.631 (0.538-0.723)	3.36	70.7	50.6

Data are reported as medians with interquartile ranges. P values comes from Mann-Whitney U test. AUCs are reported with 95% confidence interval. The selection of cut-off was based on the maximum Youden index. APNIC: arterial phase normalized iodine concentration; VPNIC: venous phase normalized iodine concentration; APNZ_eff_: arterial phase normalized effective atomic number; VPNZ_eff_: venous phase normalized effective atomic number.

In the overall cohort, the short-axis diameter of the largest lymph node has a higher AUC in predicting lymph node metastasis than the long-axis diameter (0.755 [95%CI:0.676-0.824] vs. 0.691 [95%CI:0.608-0.766]; *P*=0.004). When the short-axis diameter threshold was 6.45mm, the sum of sensitivity and specificity was the highest (62.1%and 80.7%, respectively), and the corresponding accuracy was 73.0%. Furthermore, when the long-axis diameter threshold was 8.19 mm, the sum of sensitivity and specificity was the highest (60.3% and 71.1%, respectively), and the corresponding accuracy rate was 66.7% (**Table 4**).

The median value of CEA was 3.73 (IQR: 2.17-7.58); the median value of CEA in group LNM- was 3.26 (IQR: 1.94-6.04), and the median value of CEA in group LNM+ was 4.42 (IQR: 3.08-10.83)(**Table 4**, **Figure 4C**), and the difference was significant (*P*=0.006). The AUC of CEA in predicting lymph node metastasis was 0.631 [95%CI:0.538-0.723] in all patients. When the threshold was 3.0, the accuracy was the highest (the sensitivity was 77.6% and the specificity was 45.8%), and the corresponding accuracy was 58.9%; when the threshold was 5 (abnormal clinical value), the sensitivity was 46.6%, the specificity was 68.7%, and the corresponding accuracy was 57.5%.

### Diagnostic Efficacy of DECT Quantitative Parameters for Regional Lymph Nodes in Predicting

The distributions, AUCs, cut-offs, sensitivities, and specificities of 4 DECT quantitative parameters in the overall population are shown in **Table 4**. The ROC curves of the four DECT quantitative parameters are shown in **Figure 4**. The AUC of VP NIC was significantly higher than AP normalized Z_eff_ (*P*=0.0286) and VP normalized Z_eff_ (*P*=0.0059). There was no statistical difference between VP NIC and AP NIC (*P*=0.2506).

### Radiomics Models and Their Diagnostic Power in Predicting Lymph Node Metastasis

**Figure 5:**
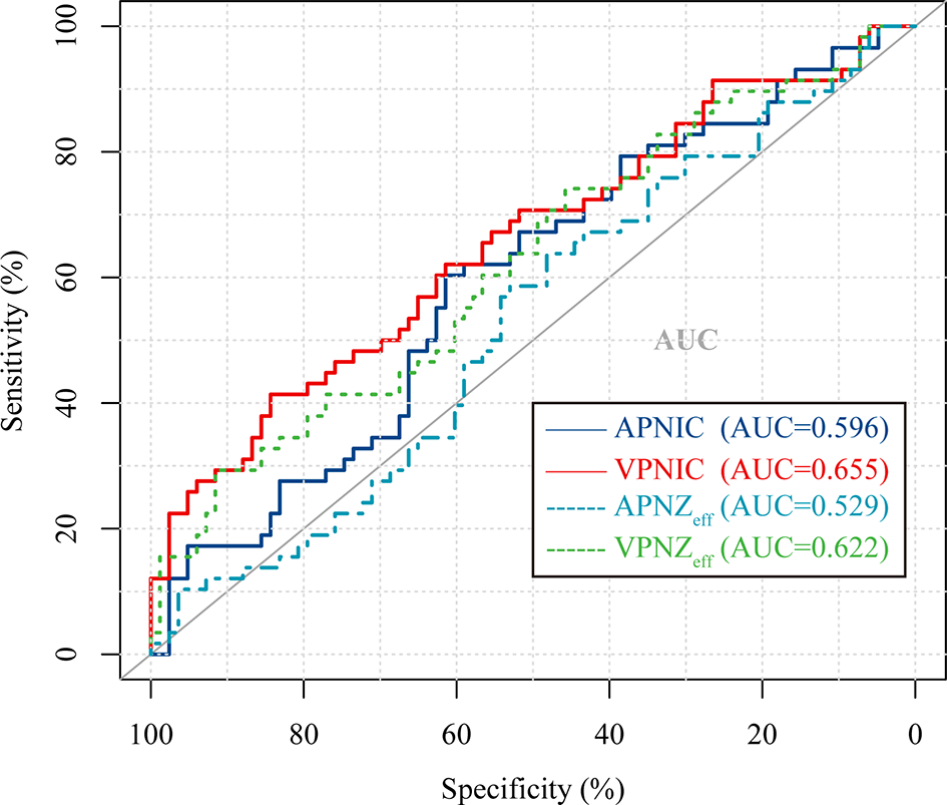
Receiver operating characteristic (ROC) curve of 4 different normalized DECT parameters used to discriminate LNM (+) from LNM (-) in the overall cohort. APNIC, arterial phase normalized iodine concentration; VPNIC, venous phase normalized iodine concentration; APNZ_eff_, arterial phase normalized effective atomic number; VPNZ_eff_, venous phase normalized effective atomic number.

After the intra-observer and inter-observer ICC tests, 424 and 542 features of the largest lymph nodes in the 120kVp-like image and the iodine map, respectively, were extracted. The results of cross-validation with different feature numbers are shown in **Supplementary Figure 1**. Rad-signature_120kVp_ is composed of 10 features (violin plots of 10 features are shown in **Supplementary Figure 2****;** coefficients of features are shown in **Supplementary Table 1**). The AUC of signatures in the training group and the testing group were 0.916 [95%CI: 0.861-0.963] and 0.922 [95%CI: 0.825-0.990], respectively (**Figure 5**).

**Figure 6:**
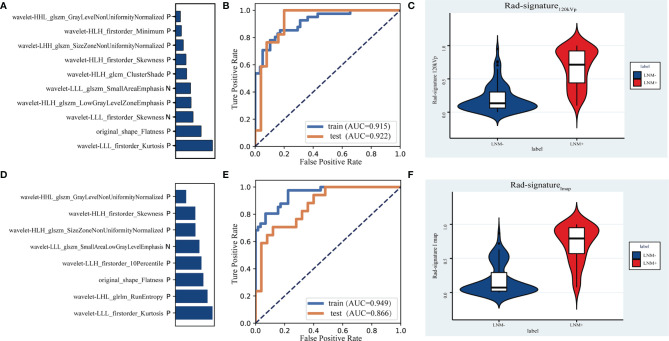
The contribution of 10 features in the signature of 120kVp-like images **(A)** and the signature of iodine map **(D)**. Receiver operating characteristic (ROC) curves to discriminate LNM (+) from LNM **(-)** for the 120kvp-like radiomics model **(B)** and iodine map **(E)** radiomics model in the training and testing cohort. Violin plots of Rad-signature_120kvp_ **(C)** and Rad-signature_Imap_ **(F)** to discriminate LNM (+) from LNM (-).

Rad-signature_Imap_ is composed of 8 features (**Supplementary Figure 2** and **Supplementary Table 2**). The AUC of signature in the training group was 0.949 [95%CI: 0.901-0.980], and the AUC in the testing group was 0.866 [95%CI: 0.742-0.961]. The feature contribution and ROC curve of the iodine map radiomics signature are shown in **Figure 6**. The cut-offs with the highest Youden index, sensitivities, specificities and IDI values of two radiomics signatures in the testing group are listed in **Table 5**. The *r* values of the Spearman correlation analysis between Rad-signature_120kvp_ and the short-axis diameter and long-axis diameter are 0.534 and 0.487, respectively, suggesting a moderate correlation (23) between Rad-signature_120kvp_ and the morphological indicators of node size.

**Table 5:** The cut-offs, sensitivities, specificities and IDI index of two radiomics signatures in the testing group.

	Rad-signature120kvp	Rad-signatureImap
cut-off	0.1851	0.5122
Sensitivity (%)	100.0 [95CI%:80.5-100.0]	70.59 [95CI%:44.0-89.7]
Specificity (%)	80.0 [95CI%:59.3-93.2]	88.00 [95CI%:68.8-97.5]
IDI to SD	0.262	0.133
IDI to LD	0.329	0.199
IDI to VPNIC	0.29	0.161
IDI to CEA	0.414	0.285

The selection of cut-off was based on the maximum Youden index. Only the IDI index to DECT quantitative parameter with the highest AUC (VPNIC) was calculated. SD, short-axis diameter; LD, long-axis diameter; VPNIC, venous phase normalized iodine concentration.

### Comparison of Different Models and Indicators in Predicting Lymph Node Metastasis

**Figure 7:**
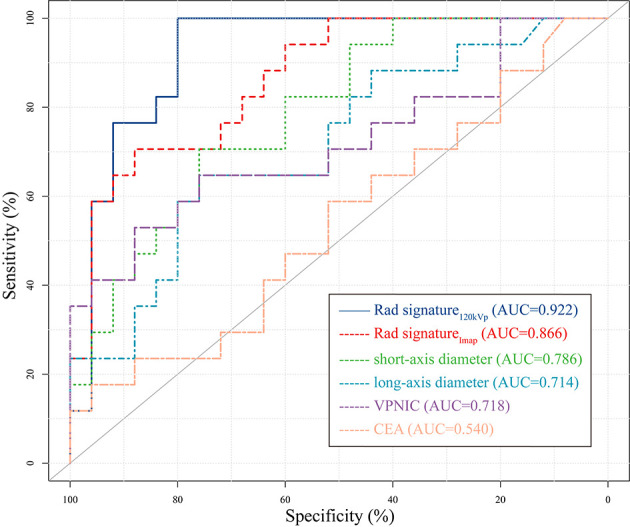
Receiver operating characteristic (ROC) curve of 6 different signatures or indicators in the testing cohort. The 120kVp-like radiomics signature had the highest area under Curve (AUC). Only the curve of the DECT quantitative parameter with the highest AUC (VPNIC) was drawn to improve readability. VPNIC: venous phase normalized iodine concentration.

The area under the curve of the six models in the testing group was compared, and Delong test results are shown in **Table 6**. Rad-signature_120kVp_ achieved the highest AUC (AUC=0.922) in prediction lymph node metastasis compared with other predictors [short-axis diameter (AUC=0.779, IDI=0.262) and long-axis diameter alone (AUC=0.714, IDI=0.329); CEA (AUC=0.540, IDI=0.414), and normalized DECT parameters (AUC=0.504~0.718, IDI=0.290-0.476)](*P*<0.05 in Delong tests). The ROC curve of 6 models is shown in **Figure 7**.

**Table 6:** *P*-values of DeLong test for AUC of 6 different signatures or indicators.

	Rad signature120kvp	Rad signatureI map	Short diameter	Long diameter	VP NIC	CEA
Rad-signature120kvp (AUC=0.922)	–	0.2299	0.0473*	0.013*	0.0359*	0.0001*
Rad-signatureImap (AUC=0.866)	0.2299	–	0.2063	0.0333*	0.1167	0.0018*
Short-axis diameter (AUC=0.779)	0.0473*	0.2063	–	0.1682	0.1719	0.0098*
Long-axis diameter (AUC=0.714)	0.013*	0.0333*	0.1682	–	0.9731	0.1049
VP NIC (AUC=0.718)	0.0359*	0.1167	0.5739	0.9731	–	0.1719
CEA (AUC=0.540)	0.0001*	0.0018*	0.0098*	0.1049	0.5739	–

*P-value<0.05; VP NIC, venous phase normalized iodine concentration.

**Figure 8:**
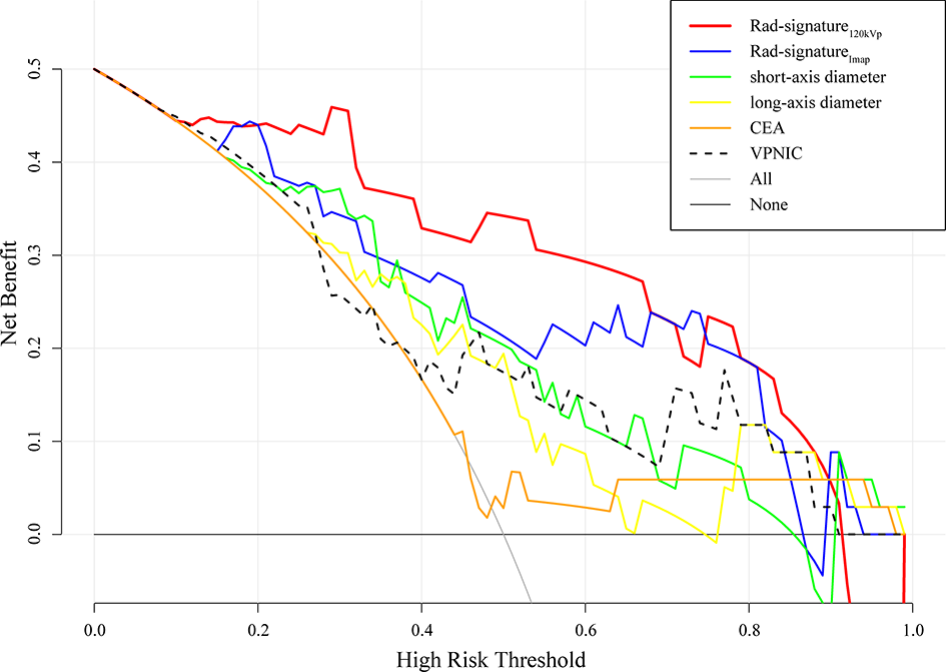
Decision curve of 6 different signatures or indicators in the testing cohort. The 120kVp-like radiomics signature had the highest area under Curve (AUC) in the majority range of risk thresholds. Only the curve of DECT quantitative parameter with the highest AUC was drawn to improve readability. VPNIC: venous phase normalized iodine concentration.

In order to evaluate the clinical practicability, a decision curve of six indicators or signatures has also been drawn (**Figure 8**). When the threshold probability was between 0 and 0.9, Rad-signature_120kvp_ had a higher net profit than other indicators and was only slightly lower than the Rad-signature_Imap_ when the threshold probability was 0.7.

## Discussion

This study explored the value of dual-energy computed tomography (DECT) radiomics of the regional largest lymph node for evaluating lymph node metastasis in patients with rectal cancer. We discovered that DECT radiomics of the regional largest short-axis lymph node could improve the prediction of lymph node metastasis in patients with rectal cancer.

The method used to determine patients’ lymph node metastasis status was based on the regional largest short-axis diameter lymph nodes. Adopting this method was based on the following: 1. In clinical practice, size is still the primary criterion for judging the status of LN (4, 8, 24). Other measures include the shape (circle) (4) and heterogeneity (8). Combined with the above criteria, the “largest short-axis diameter lymph node” was defined as the highest risk lymph node in the region. 2. Rectal cancer’s lymph node metastasis area is relatively fixed. Langman et al. (25) showed that most rectal cancer metastatic lymph nodes are located in the mesorectum close to the tumor and along the superior rectal artery. Therefore, the area defined in this study was sufficient to include metastatic lymph nodes. 3. Another reason was based on previous study data, which suggested that mapping LNs on CT may be challenging (21, 26). More lymph nodes can be seen in the specimen than on CT (26, 27). In addition, the inclusion of all lymph nodes for research has increased the number of target nodes but the uncertainty of image-specimen-pathology correspondence. We also noted that many studies adopted a method similar to ours (21, 28–30), which confirmed its feasibility. The excellent final performance of the established model also ensures the effectiveness of this method.

In this study, the predictive model based on a 120kVp-like image showed the highest diagnostic value in predicting lymph node metastasis in patients with rectal cancer. We also found that using an iodine map does not increase the extra performance compared to the 120kVp-like image reproduced by DECT. CT images combined with radiomics of the primary lesion to predict the status of lymph nodes in colorectal cancer have been reported before, showing an AUC of 0.778 (17, 30). Yet, these studies used radiomics features of primary tumors combined with clinical features (CEA levels), and CT reported the LN status. Therefore, we believe that the objective quantitative radiomics information of the lymph node itself should not be ignored.

After the preprocessing and selection, 10 and 8 features of the largest lymph nodes in the common portal phase image and the iodine map, respectively, were reserved. Flatness was the only morphological feature contained in the two imaging radiomics signatures. It represents the ratio of the long-axis length to the shortest-axis length (31); the closer it is to the spherical shape, the closer it is to 1. In other words, “round” lymph nodes tend to be malignant, which is consistent with previous studies (32) and guidelines (4).

Intensity-based statistical features shared by the iodine map and the 120kVp-like signatures included Skewness and Kurtosis. Moreover, each of the two signatures has a different histogram feature. Minimum was found in the 120kVp-like signature and 10Percentile (P10) in the iodine map signature. In fact, the meaning of the two is similar. P10 is more robust to grey level outliers than the minimum grey level (31). Skewness and Kurtosis may represent low-enhancement areas in the lymph nodes. Necrosis is the most common type of metastatic lymph node, and the low-enhancement areas in the lymph nodes indicate necrotic components in the lymph nodes. The feature Minimum and 10Percentile may indicate that the infiltration of surrounding fat may increase the density of low-density fat in the ROI. It is worth mentioning that some of the DECT quantitative parameters, such as NIC, are essentially Intensity-based statistical feature Mean based on the iodine map.

Texture features were also found to be relevant. Observing the meaning of these texture features and related conditions, the heterogeneity of metastatic lymph nodes was higher, which is also in line with our conventional cognition. Rectal benign lymph nodes include follicle, sinusoidal and mixed types, while metastatic lymph nodes include two major types of partial and complete tumor invasion and four subtypes of cellular proliferation, fibrosis, necrosis, and cyst formation (33). Thus, the heterogeneity analysis of lymph nodes is crucial. Establishing a correlation between conventional imaging findings and metastatic infiltration is challenging (25, 34), which is why size is always the main assessment parameter. The radiomics features quantify these heterogeneous manifestations to a certain extent; these features are usually not recognized and distinguished by the naked eye (16). The study found that texture features, shape, and histogram features overlap between the signature of the iodine map and the 120kVp-like image. Some features are slightly different but similar in meaning. It is generally believed that radiomics features lack repeatability, especially from different CT modalities. Nevertheless, these features remain stable in different reconstructed images. Therefore, these features should be further explored and validated. On the other hand, no feature directly reflects the size of the lymph node. Correlation analysis indicated that the *r*-value of the radiomic score and size parameter is around 0.5, which means that the radiomic signature still has a moderate correlation with the size of the lymph node.

The sensitivity and specificity of the maximum short-axis diameter of regional lymph nodes in this study were similar to those of CT reported by META analysis (34) and comparative analysis (6) but also higher than those of long-axis diameter and CEA. This shows that in the absence of radiomics or other analysis methods, short-axis diameter is still one of the most effective criteria for judging lymph node involvement of rectal cancer in clinical practice. It should be noted that, in this study, we measured the long-axis diameter of the highest-risk lymph node, which does not represent the longest diameter of all regional lymph nodes. Therefore, the diagnostic efficiency of the actual maximum longest diameter of lymph nodes in the region may be lower. In addition, because some benign lymph nodes tend to show slender type, not included by our criteria, it significantly reduces the specificity of diagnosis.

The value of DECT quantitative parameters assessing rectal cancer’s benign and malignant lymph nodes has attracted attention in recent years. However, the results from previous studies are not consistent (8, 21, 35). Nevertheless, no consensus on the diagnostic efficacy of DECT quantitative parameters in lymph node diagnosis has been proposed. Our results suggested an AUC of 0.7 when using DECT quantitative parameters alone, which is similar to data reported by Al-Najami et al. (36) but lower than others’ reports (21, 24). Based on the above results, we think that some studies may overestimate the diagnostic performance of DECT parameters. The result also confirms that the efficiency of the iodine map signature is not better than that of the 120kVp-like venous phase signature. The essence of these quantitative parameters reflects the enhanced regional iodine concentration. Metastatic lymph nodes of rectal cancer show relatively low iodine concentration due to necrosis and tumor infiltration. These concentrations can also be quantified by ordinary portal phase CT images. Yet, the factors that lead to the iodine uptake of lymph nodes may vary, such as circulatory status and lymph node blood supply. Therefore, simply measuring a lymph node’s average iodine concentration or atomic number without considering the morphological characteristics or heterogeneity of nodes does not provide sufficient diagnostic value.

Serum CEA (carcinoembryonic antigen) is one of the most important and commonly used tumor markers for rectal cancer (35). CEA is overexpressed in more than 70% of rectal cancer and can guide tumor detection and treatment (37). In this study, the accuracy of CEA to predict the N stage was only 57% when the abnormal value standard was 5ng/ml (Clinical threshold); however, the specificity of the diagnosis of lymph node metastasis was higher than the sensitivity, which was similar to data reported by Moertel et al. (38). This data indicates that lymph node metastasis can be suspected when CEA is greater than 5ng/ml, and regional lymph nodes should be more carefully evaluated. Yet, our data suggest that CEA alone may not be the best indicator of lymph node metastasis and could be jointly used with other indicators, as reported by some previous studies (17, 37).

This study still has some limitations: 1. this is a single-center study with relatively small sample size. 2. Subjects of this study are patients who have undergone surgery without neoadjuvant therapy: yet, assessing the nature of lymph nodes after neoadjuvant therapy has always been a challenging process. 3. This study did not consider some semantic features used in clinical practice such as “texture”, “border”, and “shape” for comparison. 4. This study did not discuss the combined value of some indicators and radiomics signatures. 5. The patient’s overall lymph node metastasis status is judged based on the highest-risk lymph nodes. There is no one-to-one correspondence between the pathology and the lymph nodes on the image. The model cannot distinguish the N1 or N2 stage from LNM. Previous reports showed that metastatic lymph nodes could be found in images (39), but if these small lymph nodes are the only metastatic lymph nodes of the patient, it is impossible to make a correct diagnosis using pure imaging methods. 6. Finally, the reproducibility of radiomics features has always been questioned. Further research is needed to establish a more robust model.

To sum up, a predictive radiomics model based on a 120kVp-like image and the largest short-axis diameter lymph node showed the highest diagnostic value in predicting lymph node metastasis in patients with rectal cancer and may become an effective biomarker for assessing the patient’s lymph node status in these patients. Contrary, DECT quantitative parameters and iodine maps do not provide enough information to determine the nature of lymph nodes in rectal cancer. In the absence of radiomics methods, the diagnosis should be based on an assessment of the short-axis diameter of the lymph node and subjective assessment (e.g., whether LN is round and heterogeneous).

## Data Availability Statement

The original contributions presented in the study are included in the article/**Supplementary Material**. Further inquiries can be directed to the corresponding author.

## Ethics Statement

The studies involving human participants were reviewed and approved by Ethics Committee of Affiliated Hospital of Jiangsu University. The patients/participants provided their written informed consent to participate in this study.

## Author Contributions

DW and ZZ contribute equally to this work. DW, ZZ, LZ, and SJ contributed to the conception of the study. ZZ, LZ, JZ, and QZ performed the data measurement. DW, ZZ, and SW performed the model development. ZZ, DW, and ML contributed significantly to analysis and manuscript preparation. ZZ, XM, PZ, and JX performed the data analysis and wrote the manuscript. HZ, MW, JC, XF, and XM helped perform the analysis with constructive discussions. All authors contributed to the article and approved the submitted version.

## Funding

This study was supported by the Key project of Jiangsu Provincial Health Commission (Project No.: K2019024), Natural Science Foundation of Jiangsu Province (Project No.: BK20191223), Special Funds for Roentgen Imaging Research of Jiangsu Medical Association (Project No.: SYH-3201150-0023), PhD research startup foundation of Jiangsu University Affiliated Hospital (Project No.: jdfyRC2017010), Young medical talents in Jiangsu Province (Project No.: QNRC2016831), Scientific research project of Jinshan Talent Training Program for high-level leading Talents, Postdoctoral Research Program of Jiangsu Province (Project No.: 2018K299C), and China Postdoctoral Science Foundation (Project No.: 2018M640446).

## Conflict of Interest

The authors declare that the research was conducted in the absence of any commercial or financial relationships that could be construed as a potential conflict of interest.

## Publisher’s Note

All claims expressed in this article are solely those of the authors and do not necessarily represent those of their affiliated organizations, or those of the publisher, the editors and the reviewers. Any product that may be evaluated in this article, or claim that may be made by its manufacturer, is not guaranteed or endorsed by the publisher.

## References

[1] Bray F Ferlay J Soerjomataram I Siegel RL Torre LA Jemal A. Global Cancer Statistics 2018: GLOBOCAN Estimates of Incidence and Mortality Worldwide for 36 Cancers in 185 Countries. CA Cancer J Clin (2018) 68:394–424. doi: 10.3322/caac.21492 30207593

[2] Brenner H Kloor M Pox CP. Colorectal Cancer. Lancet (2014) 383:1490–502. doi: 10.1016/S0140-6736(13)61649-9 24225001

[3] Horvat N Carlos Tavares Rocha C Clemente Oliveira B Petkovska I Gollub MJ. MRI of Rectal Cancer: Tumor Staging, Imaging Techniques, and Management. RadioGraphics (2019) 39:367–87. doi: 10.1148/rg.2019180114 PMC6438362 30768361

[4] Beets-Tan RGH Lambregts DMJ Maas M Bipat S Barbaro B Curvo-Semedo L. Magnetic Resonance Imaging for Clinical Management of Rectal Cancer: Updated Recommendations From the 2016 European Society of Gastrointestinal and Abdominal Radiology (ESGAR) Consensus Meeting. Eur Radiol (2018) 28:1465–75. doi: 10.1007/s00330-017-5026-2 PMC5834554 29043428

[5] Elsholtz FHJ Asbach P Haas M Becker M Beets-Tan RGH Thoeny HC. Introducing the Node Reporting and Data System 1.0 (Node-RADS): A Concept for Standardized Assessment of Lymph Nodes in Cancer. Eur Radiol (2021) 31:6116–24. doi: 10.1007/s00330-020-07572-4 PMC8270876 33585994

[6] Gao Y Li J Ma X Wang J Wang B Tian J. The Value of Four Imaging Modalities in Diagnosing Lymph Node Involvement in Rectal Cancer: An Overview and Adjusted Indirect Comparison. Clin Exp Med (2019) 19:225–34. doi: 10.1007/s10238-019-00552-z 30900099

[7] Al-Sukhni E Milot L Fruitman M Beyene J Victor JC Schmocker S. Diagnostic Accuracy of MRI for Assessment of T Category, Lymph Node Metastases, and Circumferential Resection Margin Involvement in Patients With Rectal Cancer: A Systematic Review and Meta-Analysis. Ann Surg Oncol (2012) 19:2212–23. doi: 10.1245/s10434-011-2210-5 22271205

[8] Brown G Richards CJ Bourne MW Newcombe RG Radcliffe AG Dallimore NS. Morphologic Predictors of Lymph Node Status in Rectal Cancer With Use of High-Spatial-Resolution MR Imaging With Histopathologic Comparison. Radiology (2003) 227:371–7. doi: 10.1148/radiol.2272011747 12732695

[9] Gröne J Loch FN Taupitz M Schmidt C Kreis ME. Accuracy of Various Lymph Node Staging Criteria in Rectal Cancer With Magnetic Resonance Imaging. J Gastrointest Surg (2018) 22:146–53. doi: 10.1007/s11605-017-3568-x 28900855

[10] Goo HW Goo JM. Dual-Energy CT: New Horizon in Medical Imaging. Kor J Radiol (2017) 18:555–69. doi: 10.3348/kjr.2017.18.4.555 PMC5447632 28670151

[11] Agrawal MD Pinho DF Kulkarni NM Hahn PF Guimaraes AR Sahani DV. Oncologic Applications of Dual-Energy CT in the Abdomen. Radiographics (2014) 34:589–612. doi: 10.1148/rg.343135041 24819783

[12] Boellaard TN Henneman ODF Streekstra GJ Venema HW Nio CY van Dorth-Rombouts MC. The Feasibility of Colorectal Cancer Detection Using Dual-Energy Computed Tomography With Iodine Mapping. Clin Radiol (2013) 68:799–806. doi: 10.1016/j.crad.2013.03.005 23615035

[13] Özdeniz İ İdilman İS Köklü S Hamaloğlu E Özmen M Akata D. Dual-Energy CT Characteristics of Colon and Rectal Cancer Allows Differentiation From Stool by Dual-Source CT. Diagn Interv Radiol (2017) 23:251–6. doi: 10.5152/dir.2017.16225 PMC5508947 28440784

[14] Chuang-Bo Y Tai-Ping H Hai-Feng D Yong-Jun J Xi-Rong Z Guang-Ming M. Quantitative Assessment of the Degree of Differentiation in Colon Cancer With Dual-Energy Spectral CT. Abdom Radiol (NY) (2017) 42:2591–6. doi: 10.1007/s00261-017-1176-6 28500383

[15] Lambin P Leijenaar RTH Deist TM Peerlings J de Jong EEC van Timmeren J. Radiomics: The Bridge Between Medical Imaging and Personalized Medicine. Nat Rev Clin Oncol (2017) 14:749–62. doi: 10.1038/nrclinonc.2017.141 28975929

[16] Gillies RJ Kinahan PE Hricak H. Radiomics: Images Are More Than Pictures, They Are Data. Radiology (2016) 278:563–77. doi: 10.1148/radiol.2015151169 PMC4734157 26579733

[17] Huang Y Liang C He L Tian J Liang C Chen X. Development and Validation of a Radiomics Nomogram for Preoperative Prediction of Lymph Node Metastasis in Colorectal Cancer. JCO (2016) 34:2157–64. doi: 10.1200/JCO.2015.65.9128 27138577

[18] Benson AB Venook AP Al-Hawary MM Arain MA Chen Y-J Ciombor KK. NCCN Guidelines Insights: Rectal Cancer, Version 6.2020. J Natl Compr Canc Netw (2020) 18:806–15. doi: 10.6004/jnccn.2020.0032 32634771

[19] Papanikolaou N Matos C Koh DM. How to Develop a Meaningful Radiomic Signature for Clinical Use in Oncologic Patients. Cancer Imaging (2020) 20:33. doi: 10.1186/s40644-020-00311-4 32357923 PMC7195800

[20] Halligan S Menu Y Mallett S. Why did European Radiology Reject My Radiomic Biomarker Paper? How to Correctly Evaluate Imaging Biomarkers in a Clinical Setting. Eur Radiol (2021) 31:9361–8. doi: 10.1007/s00330-021-07971-1 PMC8589811 34003349

[21] Yang Z Zhang X Fang M Li G Duan X Mao J. Preoperative Diagnosis of Regional Lymph Node Metastasis of Colorectal Cancer With Quantitative Parameters From Dual-Energy CT. Am J Roentgenol (2019) 213:W17–25. doi: 10.2214/AJR.18.20843 30995087

[22] Peduzzi P Concato J Kemper E Holford TR Feinstein AR. A Simulation Study of the Number of Events Per Variable in Logistic Regression Analysis. J Clin Epidemiol (1996) 49:1373–9. doi: 10.1016/s0895-4356(96)00236-3 8970487

[23] Schober P Boer C Schwarte LA. Correlation Coefficients: Appropriate Use and Interpretation. Anesth Analg (2018) 126:1763–8. doi: 10.1213/ANE.0000000000002864 29481436

[24] Liu H Yan F Pan Z Lin X Luo X Shi C. Evaluation of Dual Energy Spectral CT in Differentiating Metastatic From non-Metastatic Lymph Nodes in Rectal Cancer: Initial Experience. Eur J Radiol (2015) 84:228–34. doi: 10.1016/j.ejrad.2014.11.016 25497234

[25] Langman G Patel A Bowley DM. Size and Distribution of Lymph Nodes in Rectal Cancer Resection Specimens. Dis Colon Rectum (2015) 58:406–14. doi: 10.1097/DCR.0000000000000321 25751797

[26] Al-Najami I Beets-Tan RGH Madsen G Baatrup G. Dual-Energy CT of Rectal Cancer Specimens: A CT-Based Method for Mesorectal Lymph Node Characterization. Dis Colon Rectum (2016) 59:640–7. doi: 10.1097/DCR.0000000000000601 27270516

[27] Cui C Cai H Liu L Li L Tian H Li L. Quantitative Analysis and Prediction of Regional Lymph Node Status in Rectal Cancer Based on Computed Tomography Imaging. Eur Radiol (2011) 21:2318–25. doi: 10.1007/s00330-011-2182-7 21713526

[28] Choi J Oh SN Yeo D-M Kang WK Jung C-K Kim SW. Computed Tomography and Magnetic Resonance Imaging Evaluation of Lymph Node Metastasis in Early Colorectal Cancer. World J Gastroenterol (2015) 21:556–62. doi: 10.3748/wjg.v21.i2.556 PMC4294167 25593474

[29] Brunette LL Bonyadlou S Ji L Groshen S Shuster D Mehta A. Predictive Value of FDG PET/CT to Detect Lymph Node Metastases in Cervical Cancer. Clin Nucl Med (2018) 43:793–801. doi: 10.1097/RLU.0000000000002252 30153151 PMC7456572

[30] Chen L-D Liang J-Y Wu H Wang Z Li S-R Li W. Multiparametric Radiomics Improve Prediction of Lymph Node Metastasis of Rectal Cancer Compared With Conventional Radiomics. Life Sci (2018) 208:55–63. doi: 10.1016/j.lfs.2018.07.007 29990485

[31] Zwanenburg A Vallières M Abdalah MA Aerts HJWL Andrearczyk V Apte A. The Image Biomarker Standardization Initiative: Standardized Quantitative Radiomics for High-Throughput Image-Based Phenotyping. Radiology (2020) 295:328–38. doi: 10.1148/radiol.2020191145 PMC7193906 32154773

[32] Hulsmans FH Bosma A Mulder PJ Reeders JW Tytgat GN. Perirectal Lymph Nodes in Rectal Cancer: *In Vitro* Correlation of Sonographic Parameters and Histopathologic Findings. Radiology (1992) 184:553–60. doi: 10.1148/radiology.184.2.1620864 1620864

[33] Detry RJ Kartheuser AH Lagneaux G Rahier J. Preoperative Lymph Node Staging in Rectal Cancer: A Difficult Challenge. Int J Colorectal Dis (1996) 11:217–21. doi: 10.1007/s003840050050 8951511

[34] Bipat S Glas AS Slors FJM Zwinderman AH Bossuyt PMM Stoker J. Rectal Cancer: Local Staging and Assessment of Lymph Node Involvement With Endoluminal US, CT, and MR Imaging–a Meta-Analysis. Radiology (2004) 232:773–83. doi: 10.1148/radiol.2323031368 15273331

[35] Stiksma J Grootendorst DC van der Linden PWG. CA 19-9 as a Marker in Addition to CEA to Monitor Colorectal Cancer. Clin Colorectal Cancer (2014) 13:239–44. doi: 10.1016/j.clcc.2014.09.004 25442815

[36] Al-Najami I Lahaye MJ Beets-Tan RGH Baatrup G. Dual-Energy CT can Detect Malignant Lymph Nodes in Rectal Cancer. Eur J Radiol (2017) 90:81–8. doi: 10.1016/j.ejrad.2017.02.005 28583651

[37] Spindler BA Bergquist JR Thiels CA Habermann EB Kelley SR Larson DW. Incorporation of CEA Improves Risk Stratification in Stage II Colon Cancer. J Gastrointest Surg (2017) 21:770–7. doi: 10.1007/s11605-017-3391-4 28290141

[38] Moertel CG O’Fallon JR Go VL O’Connell MJ Thynne GS. The Preoperative Carcinoembryonic Antigen Test in the Diagnosis, Staging, and Prognosis of Colorectal Cancer. Cancer (1986) 58:603–10. doi: 10.1002/1097-0142(19860801)58:3<603::aid-cncr2820580302>3.0.co;2-k 3731019

[39] Horvat N Petkovska I Gollub MJ. MR Imaging of Rectal Cancer. Radiol Clinics North America (2018) 56:751–74. doi: 10.1016/j.rcl.2018.04.004 30119772

