# Supplementary material for: A Dual-Energy CT Radiomics of the Regional Largest Short-Axis Lymph Node Can Improve the Prediction of Lymph Node Metastasis in Patients With Rectal Cancer
Source: Front Oncol. 2022 Jun 7;12:846840. doi: 10.3389/fonc.2022.846840 (PMC9209707; doi:10.3389/fonc.2022.846840)
Supplement: Supplementary file 1 [file DataSheet_1.docx]

**SUPPLEMENTARY MATERIALS**


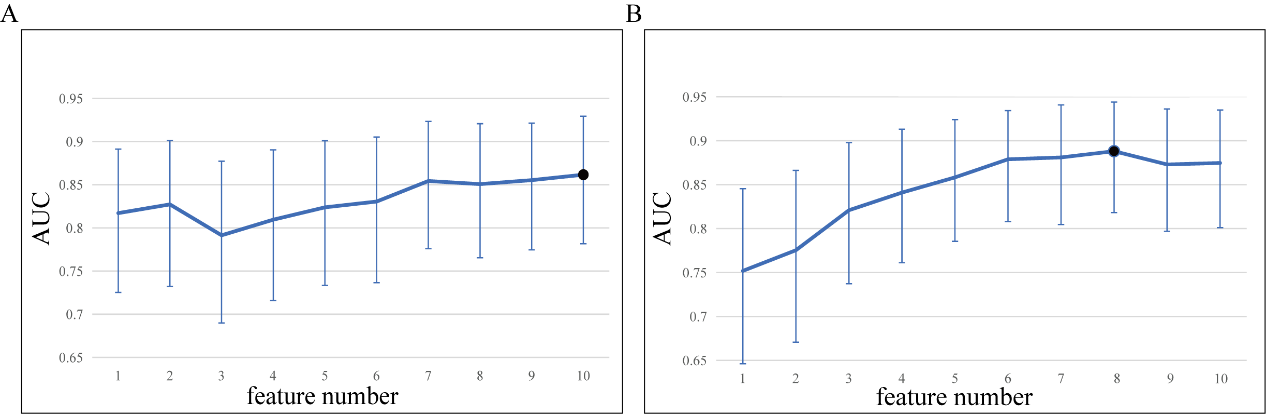


**Supplementary Figure 1.** The cross-validation result of features in 120kVp-like images **(A)** and iodine map **(B)**. Each point of the broken line represents the average AUC of the 10-fold cross-validation. Error lines are 95% confidence intervals. The black points have the highest AUC, and the corresponding features were taken as the features of the final model.


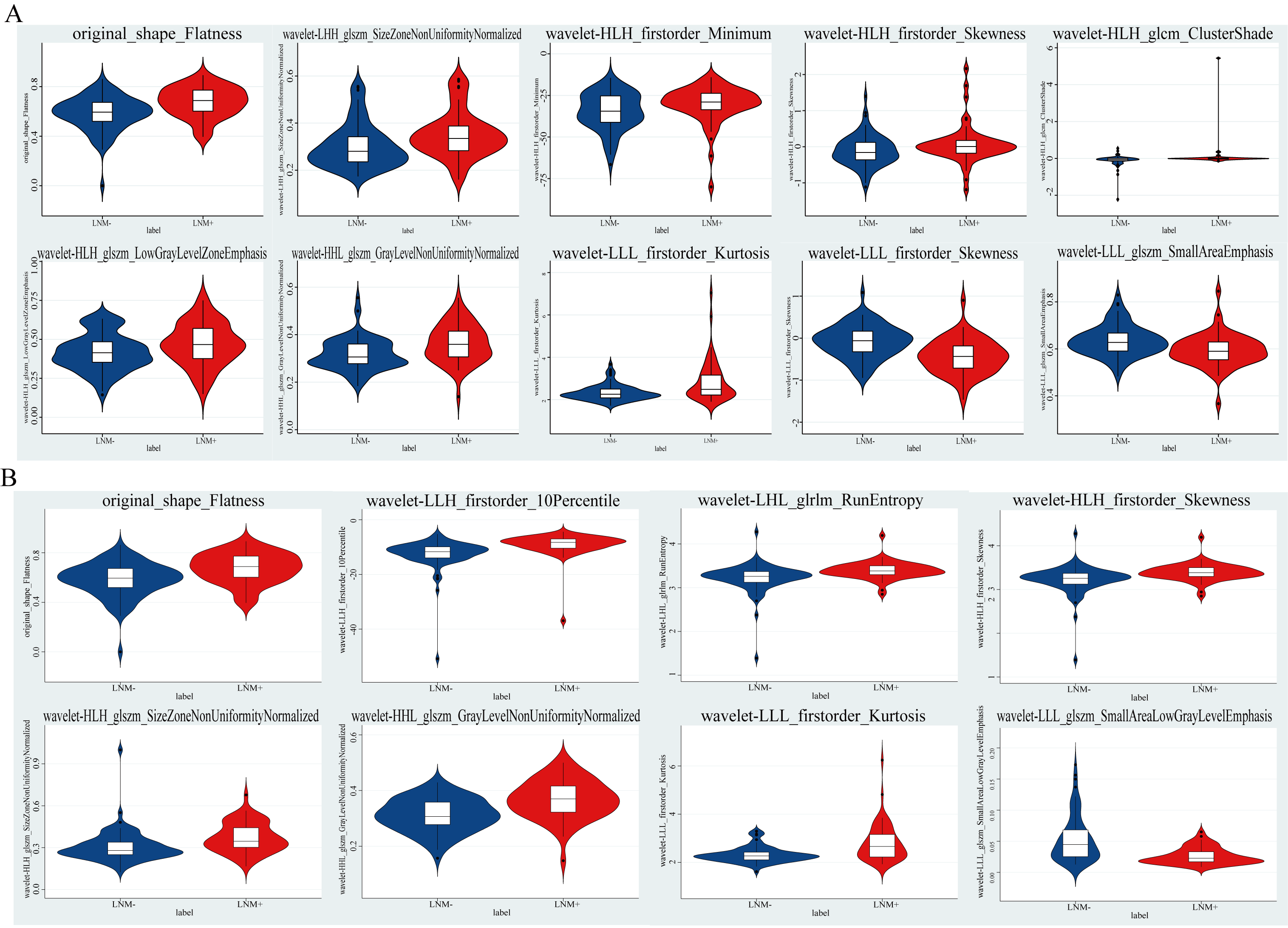


**Supplementary Figure 2**. **(A)** Violin plot of 10 features that make up Rad-signature_120kVp_ in the overall cohort. **(B)** Violin plot of 8 features that make up Rad-signature_Imap_ in the overall cohort.

| **Supplementary Table 1.** Coefficients of features in Rad-signature_120kVp_ from Logistic regression and Z-score standardization | | | |
| --- | --- | --- | --- |
|  | Coef in LR | slop in Z-score | interception in Z-score |
| original_shape_Flatness | 0.866734895 | 0.133950243 | 0.624045497 |
| wavelet-LHH_glszm_SizeZoneNonUniformityNormalized | 0.268688808 | 0.099864188 | 0.331919131 |
| wavelet-HLH_firstorder_Minimum | 0.203826104 | 9.850864528 | -32.18894451 |
| wavelet-HLH_firstorder_Skewness | 0.357046764 | 0.505266675 | -0.018092422 |
| wavelet-HLH_glcm_ClusterShade | 0.381421253 | 0.169765044 | -0.0468276 |
| wavelet-HLH_glszm_LowGrayLevelZoneEmphasis | 0.528738357 | 0.124152542 | 0.450618663 |
| wavelet-HHL_glszm_GrayLevelNonUniformityNormalized | 0.172690136 | 0.071080324 | 0.33500001 |
| wavelet-LLL_firstorder_Kurtosis | 1.246382866 | 0.80285668 | 2.592827886 |
| wavelet-LLL_firstorder_Skewness | -0.594402517 | 0.426649923 | -0.274142165 |
| wavelet-LLL_glszm_SmallAreaEmphasis | -0.509835645 | 0.063001256 | 0.615847337 |
| intercept | -0.404493285 | - | - |
| **Note:** Coef: coefficients; LR: Logistic regression | | | |

| **Supplementary Table 2.** Coefficients of features in Rad-signature_Imap_ from Logistic regression and Z-score standardization | | | |
| --- | --- | --- | --- |
|  | Coef in LR | slop in Z-score | interception in Z-score |
| original_shape_Flatness | 0.896518714 | 0.133950243 | 0.624045497 |
| wavelet-LLH_firstorder_10Percentile | 0.838100388 | 3.353931915 | -10.62425783 |
| wavelet-LHL_glrlm_RunEntropy | 1.032034655 | 0.285195071 | 3.293712699 |
| wavelet-HLH_firstorder_Skewness | 0.638415296 | 0.42606271 | 0.008343694 |
| wavelet-HLH_glszm_SizeZoneNonUniformityNormalized | 0.642972786 | 0.121006067 | 0.340924016 |
| wavelet-HHL_glszm_GrayLevelNonUniformityNormalized | 0.344389643 | 0.068690781 | 0.339226087 |
| wavelet-LLL_firstorder_Kurtosis | 1.191989357 | 0.657572602 | 2.540489349 |
| wavelet-LLL_glszm_SmallAreaLowGrayLevelEmphasis | -0.77125919 | 0.032370376 | 0.042022618 |
| intercept | -0.615350252 | - | - |
| **Note:** Coef: coefficients; LR: Logistic regression | | | |
